# Symbiotic Gene Activation is Interrupted by Endocrine Disrupting Chemicals

**DOI:** 10.1100/tsw.2001.359

**Published:** 2001-11-13

**Authors:** Jennifer E. Fox, Matthew E. Burow, John A. McLachlan

**Affiliations:** Environmental Endocrinolgy Laboratory, Center for Bioenvironmental Research at Tulane University, Tulane University Medical School, New Orleans, LA 70112, USA

**Keywords:** endocrine disruption, symbiosis, phytochemical, estrogen receptor, nitrogen fixation

## Abstract

Endocrine disrupting chemicals (EDCs) include organochlorine pesticides, plastics manufacturing by-products, and certain herbicides[1]. These chemicals have been shown to disrupt hormonal signaling in exposed wildlife, lab animals, and mammalian cell culture by binding to estrogen receptors (ER-α and ER-β) and affecting the expression of estrogen responsive genes[2,3]. Additionally, certain plant chemicals, termed phytoestrogens, are also able to bind to estrogen receptors and modulate gene expression, and as such also may be considered EDCs[4]. One example of phytoestrogen action is genistein, a phytochemical produced by soybeans, binding estrogen receptors, and changing expression of estrogen responsive genes which certain studies have linked to a lower incidence of hormonally related cancers in Japanese populations[5]. Why would plants make compounds that are able to act as estrogens in the human body? Obviously, soybeans do not intentionally produce phytoestrogens to prevent breast cancer in Japanese women.

One reason legumes, such as soybean and alfalfa, produce these phytochemicals is to initiate a symbiotic relationship with *Rhizobium* soil bacteria which are able to inhabit plant roots and, in exchange for a carbon food source from the plant, reduce 120 million tons of atmospheric nitrogen per year into ammonia, which serves as a natural fertilizer for host plants[6]. The symbiosis or cooperation between these two organisms is initiated and maintained by phytochemical signaling from the host plant to soil bacteria[7]. The exchange of chemical signals either within a cell, between cells, or even between organisms is the cellular basis of communication. Another example of chemical communication is estradiol signaling through the estrogen receptors to induce expression of estrogen responsive genes resulting in a variety of estrogen-induced cellular changes[1]. Similarly, phytochemical signaling from a host plant such as alfalfa, in the form of the phytochemicals luteolin and apigenin, is recognized by the bacterial symbiotic partner *Sinorhizobium meliloti's* NodD protein which is a transcriptional activation protein that turns on expression of key nodulation (nod) genes within the bacteria that are necessary for establishing symbiosis[8,9].

In light of the similarities between estrogenic and symbiotic signaling, our research considered the possibility that chemicals that disrupt estrogenic signaling in mammals, EDCs, also disrupt phytochemical signaling between the host plant alfalfa and the soil bacteria *Sinorhizobium meliloti* (*S. meliloti*) necessary for nitrogen-fixing symbiosis[2,3]. Our recent study published in the journal *Nature*, tested the ability of a variety of EDCs to negatively affect expression of *nod* genes in *S. meliloti*[12]. Using a reporter gene in *S. meliloti*, we quantified the amount of *nod* gene activation in response to the natural ligand, luteolin, both in the absence and the presence of a variety of EDCs[13]. Chemicals such as the insecticides pentachlorophenol and methyl parathion as well as herbicides such as 2, 4-D and 2,4,5-T (at concentrations of 50 μM each) were able to reduce *nod* gene expression from 100% (seen in untreated + luteolin) to 10%, 12%, 68%, and 63%, respectively, even in the presence of luteolin[12]. In support of the idea that these EDCs are competing for signaling with the natural ligand, when an increasing amount of the agonist luteolin was added to treated *S. meliloti*, *nod* gene activation was restored to 100% activity[12]. Other experiments measuring *in vivo* signaling, in which alfalfa sprouts had been inoculated with bacteria in the absence or presence of these inhibitory EDCs, confirmed that the presence of some EDCs reduced the communication between plant and bacteria necessary for initiating symbiosis[12]. Endocrine disrupting chemicals such as the insecticides DDT, pentachlorophenol, and methyl parathion as well as the herbicides 2,4-D and 2,4,5-T, and the plasticizer Bisphenol A were all able to disrupt signaling between phytochemicals and the NodD transcriptional activator resulting in a inhibition of *nod* gene activation both *in vitro* and *in vivo*[12].

EDCs and pollutants present in agricultural soil might be inhibiting the expression of key *nod* genes resulting in a lowering of symbiotic signaling between host plants, including important crops such as soybean and alfalfa, and soil bacteria that would normally fix nitrogen into fertilizer for the host plant[14,15]. Nitrogen-fixing symbiosis is a crucial environmental process. With the world's population at six billion and expected to double in size by the year 2050, insuring adequate crop yield through renewable natural fertilizer sources is a clear priority[10]. In agricultural regions with sufficient precipitation, nitrogen supply is the most important limiting factor for crop yield[11]. If symbiosis is adversely affected by these EDCs on a large scale, the consequences could be reduced symbiosis, loss of naturally available fixed nitrogen, and reduced crop yield in treated fields. Further implications of this study are that these pollutants, pesticides, and other EDCs which have been reported to affect endocrine signaling in organisms from single cells to exposed wildlife now have a new target in symbiotic signaling between plants and nitrogen-fixing bacteria.
